# Carbon monoxide poisoning in Denmark with focus on mortality and factors contributing to mortality

**DOI:** 10.1371/journal.pone.0210767

**Published:** 2019-01-17

**Authors:** Carsten Simonsen, Kristinn Thorsteinsson, Rikke Nørmark Mortensen, Christian Torp-Pedersen, Benedict Kjærgaard, Jan Jesper Andreasen

**Affiliations:** 1 Department of Cardiothoracic Surgery, Aalborg University Hospital, Aalborg, Denmark; 2 Department of Clinical Medicine, Aalborg University, Aalborg, Denmark; 3 Unit of Epidemiology and Biostatistics, Aalborg University Hospital, Aalborg, Denmark; 4 Biomedical Research Laboratory, Aalborg, Denmark; University of Missouri Health Care, UNITED STATES

## Abstract

**Introduction:**

Carbon monoxide (CO) poisoning is frequent worldwide but knowledge regarding the epidemiology is insufficient. The aim of this study was to clarify the extent of this intoxication, its mortality and factors associated with mortality.

**Materials and methods:**

National databases from Statistics Denmark were used to identify individuals who suffered from CO-poisoning during 1995–2015, as well as information regarding co-morbidities, mortality and manner of death.

**Results:**

During the period from 1995 to 2015, 22,930 patients suffered from CO-poisoning in Denmark, and 21,138 of these patients (92%) were hospitalized. A total of 2,102 patients died within the first 30 days after poisoning (9.2%). Among these, 1,792 (85% of 2,102) were declared dead at the scene and 310 (15% of 2,102) died during hospitalization. Deaths due to CO-poisoning from smoke were intentional in 6.3% of cases, whereas deaths due to CO containing gases were intentional in 98.0% of cases. Among patients who survived >30 days, there was no significant difference in survival when comparing hyperbaric oxygen therapy (HBO) treatment with no HBO treatment after adjustment for age and co-morbidities such as drug abuse, psychiatric disease, stroke, alcohol abuse, arterial embolism, chronic obstructive pulmonary disease, cerebrovascular disease and atrial fibrillation. Several co-morbidities predicted poorer outcomes for patients who survived the initial 30 days.

**Conclusions:**

Poisoning from smoke and/or CO is a frequent incident in Denmark accounting for numerous contacts with hospitals and deaths. Both intoxication and mortality are highly associated with co-morbidities interfering with cognitive and physical function. Treatment with HBO was not seen to have an effect on survival.

## Introduction

Since carbon monoxide (CO) has no smell, no taste and no color, it is very hard to detect without the use of a CO-detector. This stealthiness, along with its highly toxic properties, makes it extremely dangerous, and victims might not suspect any danger until they are incapacitated and need help to survive.

Persisting or late onset of neurological symptoms frequently contribute to morbidity [1–2], but arrhythmias and cardiac insufficiency are also reported following CO-poisoning [3–4].

Previously, town gas containing CO used for residential heating and CO in car exhaust were prominent causes of CO-poisoning. However, since CO has been removed from town gas in Europe and cars are equipped with catalytic converters, the number of cases has dropped markedly [5–7]. Today, smoke from fires, indoor charcoal burning, defective domestic heat sources and suicide/suicide attempts are frequent causes of CO-poisoning [8].

Different harmful mechanisms contribute to the high level of toxicity. Most commonly known is the strong binding to hemoglobin facilitated by the high affinity for the iron ions in this molecule [9]. The oxygen-carrying capacity is thus diminished, leading to universally impaired oxygen delivery. However, CO also interferes with mitochondrial enzyme activity, inhibiting aerobic metabolism [10]. Additionally, several other toxic effects occur [8]

Treatment of CO-poisoning has not evolved much during the last 50 years. It primarily consists of supplementary oxygen therapy and symptomatic treatment. Normobaric (NBO) or hyperbaric (HBO) oxygen can be administered in a pressurized chamber which is usually reserved for more severe cases. However, some patients may be too unstable to transfer to an HBO-capable center.

Although it is well documented that HBO therapy can reduce the HbCO half-life [11], the advantages of HBO therapy are still questioned due to a lack of decisive evidence of improved survival and reduced morbidity [9,12–15].

In 2003, an updated report from the Danish Health Institute collected data on all CO-poisoning cases from 1997–2001 [16]. A total of 3,671 cases were identified. This report was used for national guideline purposes only. Overall, no comprehensive epidemiological studies of CO poisoning in Denmark exist. The aim of this study was to clarify the extent of this intoxication, its mortality and factors contributing to mortality.

## Material and methods

The study was accepted by the Data Protection Agency (GEH-2014-013 I-Suite nr: 02731) and it is a retrospective nationwide cohort study. All citizens in Denmark are equipped with a unique personal identification number (CPR number), which is registered in all contacts with the health care system. Settlement of the correct rate of reimbursement is based on the correct registration of both CPR number and diagnoses. According to current legislation in Denmark, it is not necessary to obtain approval by the individual patient or general ethics approval for register-based studies. All contacts are registered in various databases administered by Statistics Denmark. These databases can be accessed for health management purposes and epidemiological research. The CPR number is encrypted in the same way across databases, which enables cross-referencing without accessing personally identifiable information. Data were accessed using encrypted remote access to Statistic Denmark´s servers. Three different databases managed by Statistics Denmark were used. The Danish National Patient Register (DNPR) has registered all hospital admissions and discharge coding diagnoses in Denmark since 1978. Diagnoses and procedures are coded according to the International Classification of Diseases (8th revision (ICD-8) through the end of 1993, and 10th revision (ICD-10) thereafter) [17]. Since 1875, the the Danish Register of Causes of Death has registered all deaths and cause(s) of deaths among citizens that died in Denmark. Registration has been in accordance with WHO’s rules since 1994 by ICD-10 [18] and The National Prescription Registry which contains information regarding all claimed prescriptions since 1994, including date of dispensation, strength and quantity. This database has been proven to contain valid information regarding the use of medication in Denmark [19].

Data from 01.01.1995 to 31.12.2015 were included in this study. We chose 01.01.1995 as the cut-off day beacause ICD coding prior to this date was different.

From the DNPR, all patients in contact with emergency departments or admitted to a hospital with the International Statistical Classification of Diseases and Related Health Problems codes (ICD-10) T58; toxic effect of carbon monoxide, and T59; Toxic effect of other gases, fumes and vapors were identified. Patients with the following irrelevant intoxications were excluded: T590; nitrogen oxides, DT591; sulfur dioxide, T592; formaldehyde, T593; lacrimogenic gas, T594; chlorine gas, T595; fluorine gas and hydrogen fluoride, T596; hydrogen sulfide, and T597; CO_2_. Co-morbidities identified in the DNPR were drug abuse, psychiatric disease, stroke, alcohol abuse, heart failure, renal failure, arterial embolism, chronic obstructive pulmonary disease (COPD), myocardial infarction, cerebrovascular disease, atrial fibrillation, and all types of dementia. Information regarding the identification of patients suffering from diabetes mellitus was extracted from The National Prescription Registry using insulin drugs as a marker (ATC code A10A).

Only patients who had contact with emergency departments or were admitted to hospitals are represented in the DNPR. To identify those patients who were declared dead at the scene the Danish Register of Causes of Death was used with the same ICD-10 criteria.

### Statistical analysis

We used the chi-square test to compare variables for patients admitted to the hospital versus patients who died at the scene and to evaluate differences between patients who received HBO and those who did not. For age, we used independent sample t-tests. A linear regression model was used to predict any trends in the number of deaths.

To minimize the effects of selection bias in our survival analysis, we used data from patients who survived for at least 30 days after poisoning in our models. To evaluate survival among patients who reached a hospital, Kaplan-Meier estimates were used, comparing the survival of patients not treated with HBO to that of patients treated with HBO. The overall difference was estimated using the log-rank test. To evaluate the effect of various co-morbidities, a Cox regression model was constructed using only data from patients who survived beyond the first 30 days after CO-poisoning. The Results are presented as hazard ratios (HR) with corresponding confidence intervals (CI). We tested for any violation of the proportional hazards assumption using Schoenfeld residuals. Age was divided into four subgroups equivalent to the quantiles: age group 1 = 0–24.4 years, age group 2 = 24.4–36.6 years, age group 3 = 36.6–50.75 years, and age group 4 = 50.8+ years. Age group 2 was used as a reference in the model.

For data management, the software SAS (version 9.4, SAS Institute, Cary, North Carolina, USA) was used. For all of the statistical analyses, the open source freeware program R was used (version 3.4.1).

## Results

**Fig 1:**
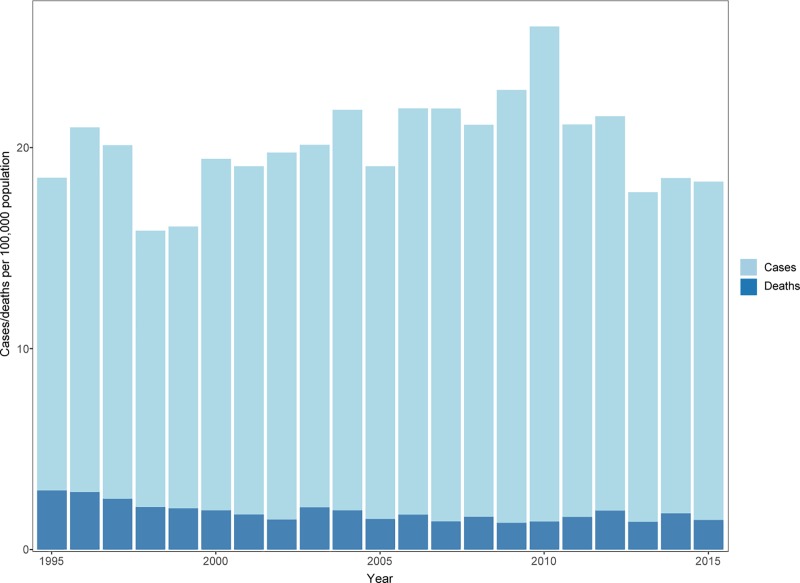
Overview of the total rate of cases and the rate of deaths by year. Total number of cases = 22,930.

A total of 22,930 patients suffered from CO-poisoning from 1995–2015 in Denmark (Fig 1). A total of 2,102(9.2%) patients died within the first 30 days after CO-poisoning directly or with CO-poisoning as a contributing cause of death. Out of this group, 1,792 (85.3%) patients were declared dead at the scene and thus never reached a hospital. Of the 21,138 patients who were hospitalized or in contact with an emergency department 310(1.5%) died within 30 days. Autopsies were performed in 40.0% of deaths after 2002. No data prior to 2002 were available. The median follow-up time in this study was 8.1 years.

Demographic characteristics of patients hospitalized with CO-poisoning and patients who died elsewhere are presented in Table 1. The mean age of the victims who died at the scene was 52.6 years (95% confidence interval (CI): 51.7–53.4 years) vs. 39.3 years (95% (CI): 39.0–39.6 years) for the hospitalized patients (p<0.001). The majority of victims were males, especially among those who did not reach a hospital (77.4%). Further comparisons of victims who died on scene vs. those who reached hospital alive revealed a larger proportion of patients who were diagnosed with alcohol abuse (21.8% vs. 12.2%, p<0.001), drug abuse (17.4% vs. 10.3%, p<0.001) and psychiatric disease (12.9% vs. 8.3%, p<0.001).

**Table 1:** Overview of sex, age and co-morbidities in various subgroups of the study population.

Variable	Group			Treatment		
	Dead at Scene	Hospitalized	p-value	No HBO	HBO	p-value
Mean age (SD)	52.6 (18.9)	39.3 (20.2)	< 0.001	39.1 (20.2)	45.2 (21.1)	< 0.001
Sex	1387(77.4)	12503(59.1)	<0.001	12229(59.1)	274(63.6)	< 0.001
30 days Mortality	1792(100.0)	310(1.5)	< 0.001	262(1.3)	48(11.1)	< 0.001
Drug Abuse	312(17.4)	2169(10.3)	< 0.001	2087(10.1)	82(19.0)	< 0.001
Alcohol Abuse	391(21.8)	2585(12.2)	< 0.001	2495(12.0)	90(20.9)	< 0.001
Psychiatric Disease	232(12.9)	1747(8.3)	< 0.001	1677(8.1)	70(16.2)	< 0.001
Stroke	92(5.1)	701(3.3)	< 0.001	675(3.3)	26(6.0)	<0.01
Heart Failure	55(3.1)	402(1.9)	< 0.001	395(1.9)	7(1.6)	0.8
Renal Failure	15(0.8)	71(0.3)	<0.01	66(0.3)	5(1.2)	<0.01
Arterial Embolism	110(6.1)	819(3.9)	< 0.001	790(3.8)	29(6.7)	<0.01
COPD	92(5.1)	643(3.0)	< 0.001	630(3.0)	13(3.0)	1.00
Myocardial Infarction	62(3.5)	433(2.0)	< 0.001	427(2.1)	6(1.4)	0.42
Cerebrovascular Disease	47(2.6)	365(1.7)	<0.01	347(1.7)	18(4.2)	< 0.001
Atrial Fibrillation	56(3.1)	422(2.0)	<0.01	405(2.0)	17(3.9)	<0.01
Other Dementia	16(0.9)	137(0.6)	0.28	131(0.6)	6(1.4)	0.10
Sclerosis	6(0.3)	44(0.2)	0.40	44(0.2)	0	0.67
Pulmonary Embolism	7(0.4)	40(0.2)	0.12	39(0.2)	-	-
Peripheral Arterial Disease	54(3.0)	203(1.0)	< 0.001	199(1.0)	-	-

Those who died at the scene vs. those where hospitalized (second column). In hospitalized patients; those who were treated with hyperbaric oxygen (HBO) therapy vs. those who were not (third column). COPD = chronic obstructive pulmonary disease.

**Fig 2:**
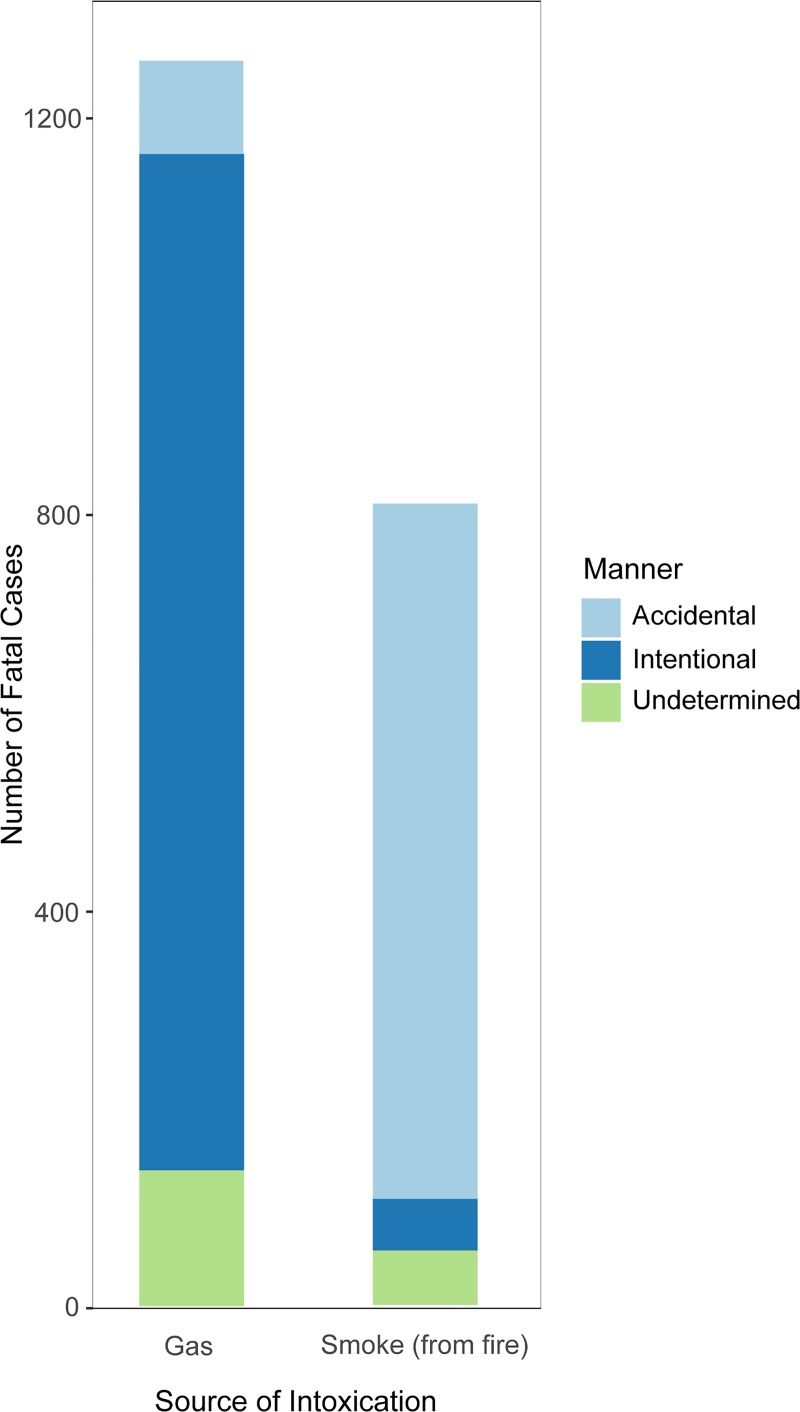
Number of fatalities by type of exposure and manner of death.

**Fig 3:**
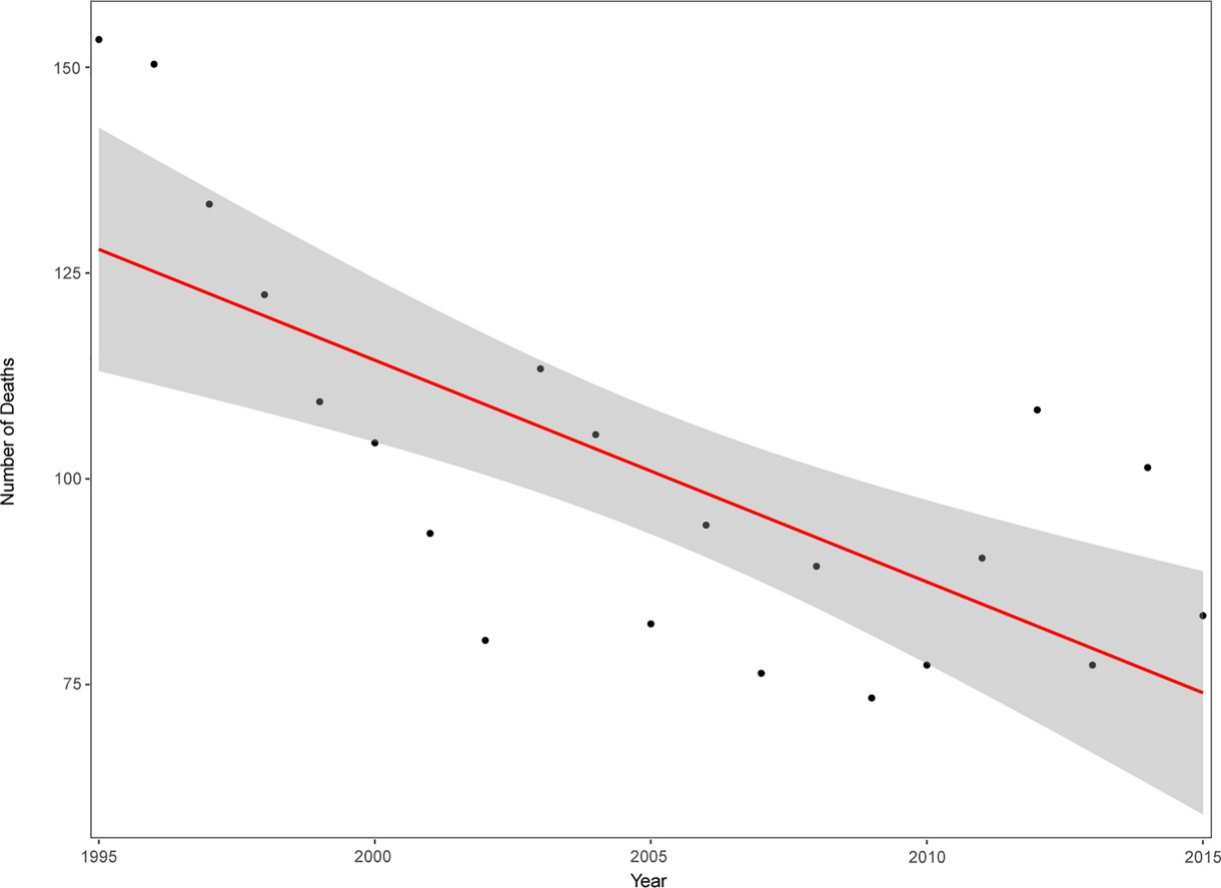
Trend in the number of fatal cases. The grey area depicts 95% CI, p<0.001.

Co-morbidities among initial surviving patients receiving HBO therapy (n = 431) were different from those who did not receive HBO therapy (Table 1). The mean age in the HBO group was higher (45.2 years vs. 39.1 years, p<0.001), and there was a higher 30-day mortality rate (11.1% vs. 1.3%, p<0.001). A higher proportion of patients in the HBO group had been diagnosed with alcohol abuse (20.9% vs. 12.0%, p<0.001), drug abuse (19.0% vs. 10.1%, p<0.001) and psychiatric disease (16.2% vs. 8.1%, p<0.001). A greater proportion were males in both the HBO group (59.1%) and in the no HBO group (63.6%) and the difference between these groups was significant (p<0.001). Out of 825 deaths due to CO-containing smoke from fire/flames, 718 were accidental (87.0%), 52 were intentional (7.2%) and 55 were undetermined (6.7%) (Fig 2). Out of 1,132 deaths due to gas/vapors, 94 were accidental (8.3%), 1,025 were intentional (91.1%) and 13 were undetermined intent (1.1%). A total of 137 died of other forms of CO-exposure with undetermined manner of death. There was a significant trend over time towards a decreasing number of deaths due to CO-poisoning (R^2^ = 0.49, p<0.001) (Fig 3).

**Fig 4:**
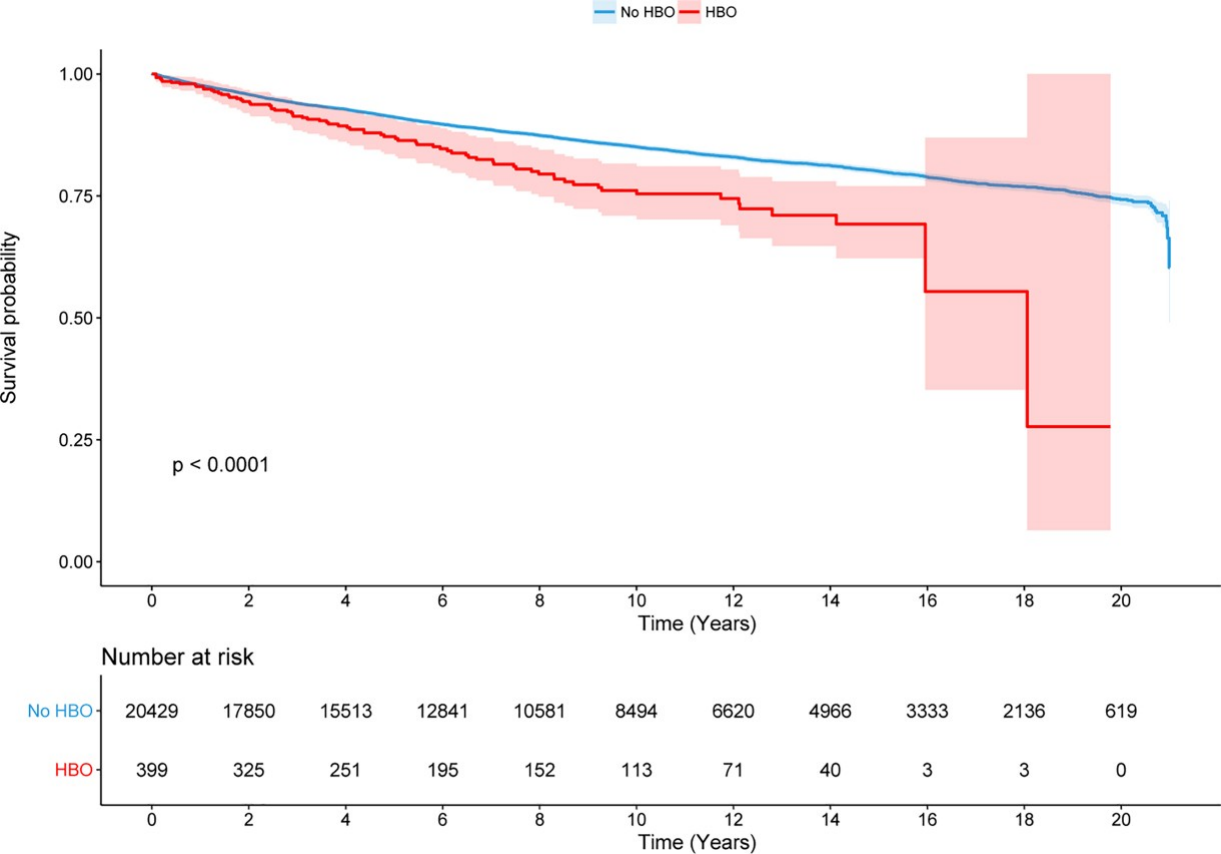
Kaplan-Meier plot of survival depending on treatment. Only those who lived beyond 30 days are included. Light blue-/red-colored areas depicts 95% CI.

**Fig 5:**
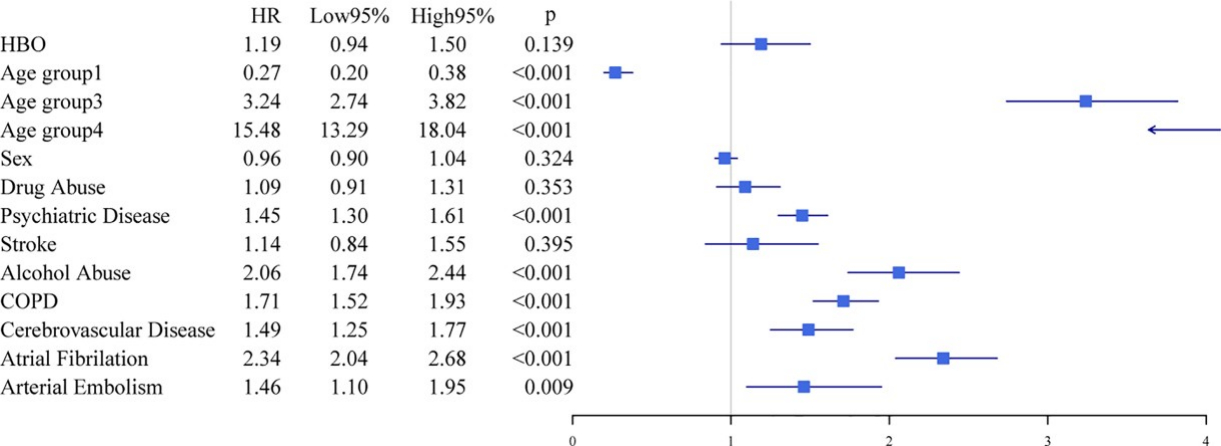
Forest plot for the Cox proportional hazards model. Age group 1 = 0–24.4 years, age group 2 (reference) = 24.4–36.6 years, age group 3 = 36.6–50.75 years, and age group 4 = 50.8+ years. COPD = Chronic Obstructive Pulmonary Disease. Lines illustrate 95% CI.

A Kaplan-Meier plot illustrates the survival of hospitalized patients who survived >30 days (Fig 4). This plot demonstrates diverging survival lines with inferior survival among patients treated with HBO (p<0.001). Significant differences were observed already 6 years. After adjustment for co-morbidities in a Cox regression model, HBO treatment was not independently associated with increased mortality (HR = 1.2, p = 0.14) (Fig 5). Age was significantly associated with the risk of mortality. The HR increased with increasing age; age group 3 had an HR = 3.2, p<0.001, and age group 4 had an HR = 15.5, p<0.001. Other factors associated with increased mortality were alcohol abuse (HR = 2.1, p<0.001), psychiatric disease (HR = 1.5, p<0.001), arterial embolism (HR = 1.5, p = 0.01), COPD (HR = 1.7, p<0.001), cerebrovascular disease (HR = 1.5, p<0.001) and atrial fibrillation (AF) (HR = 2.3, p<0.001). Drug abuse (HR = 1.1, p = 0.35), stroke (HR = 1.1, p = 0.40), and gender (HR = 0.96, p = 0.32) were not significant predictors of mortality.

## Discussion

This study shows that HBO treatment was not associated with survival benefit compared with that of no HBO treatment after adjustment for several confounding factors. It also demonstrated that a large percentage of CO-poisoned victims had a history of psychiatric diseases and alcohol abuse. These co-morbidities, along with increasing age, were strongly associated with mortality. Approximately, 50% of deaths were intentional; however, a higher proportion of deaths caused by CO-poisoning from gas were intentional compared to intentional deaths due to poisoning from smoke from fire.

There is a strong association between suicide and psychiatric disease as well as between suicide and the abuse of alcohol and drugs [20–21]. This is clearly reflected in our study as a large proportion of deaths from CO-poisonings were suicides (51%), and a substantial number of patients had psychiatric disease or, abused alcohol and drugs (Table 1). Among contributing factors, victims may have a higher risk of exposure to CO-poisoning since impaired cognitive function can cause unsafe behavior in connection with potential sources of CO, e.g., fire and gas installations. It is also plausible that in cases of accidental exposure, this group may not be as capable of escaping outside as quickly as other poisoning victims, resulting in increased severity of intoxication. These effects could also be the case for older victims and victims with a prior history of cerebrovascular disease, stroke and AF. The co-morbidities may also explain why the average lifespan after CO-poisoning seems to be reduced (Fig 4) when compared to the general life expectancy in Denmark, which is approximately 80 years. These findings are consistent with a previous study that showed increased long-term mortality among survivors of acute CO-poisoning who received HBO treatment [22].

There was a significant difference in long-term survival among patients receiving treatment with HBO therapy versus no HBO therapy in our survival analysis (p>0.001). These findings are also consistent with the results of the above-mentioned study by Hampson et al. [22]. In our study, HBO treatment was not an independent predictor of mortality after adjustments in the Cox regression model. The difference in long-term survival is therefore not caused by HBO therapy itself. One plausible explanation could be the significant difference in the burden of comorbidities between the groups.

The overall 30-day mortality was relatively high (9.2%), including those who died at the scene. However, among those who arrived at the hospital, the 30-day mortality was only 1.5%. This difference may be explained by the fact that a large proportion of CO victims were declared dead at the scene and therefore did not contribute to the in-hospital 30-day mortality. It may also be speculated that a person trying to commit suicide from CO poisoning will be discovered later, in a more poisoned condition or dead, than those who become intoxicated by accident. Furthermore, transportation to the hospital may be a marker for a patient with an inherently better prognosis. A number of factors may contribute to selection bias of the patients receiving HBO therapy. There is no international consensus regarding the criteria for treatment with HBO therapy [23]. In Denmark, HBO therapy is primarily performed at a single hospital (Rigshospitalet, Copenhagen) although a hyperbaric chamber also exists at one other university hospital. The referral criteria for hyperbaric oxygen (HBO) therapy in Denmark are neurological abnormities that are more severe than normal head-aches, present or previous loss of consciousness, cardiac arrhythmias/depression, pregnancy and carboxyhemoglobin (HbCO) more than 25%, regardless of other symptoms [24]. The criteria for HBO therapy in Denmark tend to select patients with a higher degree of severity, while logistic issues with transportation of critically ill patients from other parts of Denmark may tend to exclude some of the most severe cases. Additionally, it should be noted that the vast majority of patients who died, had no opportunity for receiving HBO treatment, as they were declared dead on scene. By looking only at the patients who survived >30 days after admission, we can eliminate some of this bias. However, to evaluate the true difference between HBO versus NBO therapies, more prospective randomized studies are required, as previous studies of this character have not sufficiently uncovered the role of HBO therapy [13].

It is interesting to note that the percentage of patients receiving HBO therapy was less than 2%; it has been reported that this treatment was offered to a higher percentage of patients in Taiwan [14]. This difference may be explained by differences regarding access to hospitals for patients with only minor CO-poisoning. The differences regarding access to HBO treatment may explain differences in survival among studies.

Only a few Danish epidemiologic studies regarding CO-poisoning exist, and most of these studies have a forensic focus. In a paper from 1960, Dalgaard examined deaths related to town gas [5] and identified a total of 407 cases (299 suicides, 90 accidents, 18 homicides). This study did not include other cases of CO-poisoning. In 2007, Thomsen and Gregersen investigated CO-related deaths from 1995–1999 and found that 22 cases of town gas exposure resulted in death out of a total of 449 non- fire related CO-poisoning cases [6]. However, these studies are not representative of the current conditions because town gas was phased out from 1989–2007. Another study by Thomsen and Gregersen published in 2005 [7] looked at all suicides caused by CO-poisoning from car exhaust gas from 1995–1999. A total of 388 cases were identified, constituting 9.3% of all suicides in Denmark during that period. The catalytic converter has been mandatory in all new vehicles sold in Denmark since 1990. However, since this only applied to new cars at that time, the real implementation has been over a period of 10–20 years. This probably plays a role in the decrease in the number of CO-poisoning cases in the period from 1987–1999 and could be a result of an increased number of cars operating with catalytic converters [7]. This is supported by a study by Hampson and Holm from 2015 [25]. The authors compared CO emission data with cases of CO poisoning. They concluded that there was a strong association between the reduction in vehicle exhaust CO emissions and the decreasing numbers of CO poisoning cases from 1985—this is likely to be a result of the introduction of the catalytic converters in 1975.

In an American study from 2007, the total number of emergency department visits due to CO-poisoning was estimated to be approximately 50,000 annually [26]. The authors reached this number by extrapolating data from 2005 from five states. This was equivalent to 16 cases of CO-poisoning per 100,000 inhabitants/year. The corresponding number of cases in 2005 in the present study was 19/100,000 per year. These similar incidences probably reflect the comparable daily living conditions in western countries and comparable methods of registration. Another American paper from 2016 identified the number of deaths from CO-poisoning by using national data from death certificates [27]. The number of fatalities in the US in 2005 was 1,702 with a significant trend for a decreasing number of deaths from 1999 to 2014. These findings are in agreement with the results of the present study.

In a paper published in 2012 [28], the authors gained information regarding CO-poisoning and the associated mortality from 28 European WHO member states. During the period from 1980 to 2008, they reported 140,490 CO-related deaths in total, with a death rate of 2.2 per 100,000 people annually. However, the range was between 0.02 and 12.8 depending on the country. Six countries provided additional data regarding admissions due to CO-poisoning, with a total of 31,473 admissions. The mean rate of admissions was 2.33 per 100,000 people/year. This paper identified males as more prone to suffer from CO-poisoning and established age as a predictor of increased mortality. These findings are consistent with the findings in the present study. In contrast with the findings of the present study other studies suggest that HBO therapy is associated with lower short- and long-term mortality [12,14]. The reason for this difference may be differences in treatment indication, differences in logistics and differences regarding patient population and database registrations. The primary strength of the present study is the completeness of data from validated databases. Furthermore, Denmark is a small country with a high degree of homogeneity in the population as well as in health care services, which are completely free of charge for all citizens. The state reimburses the hospital for the cost of each patient, and since reimbursement is dependent on correct coding, we assume that this adds to the credibility of the data.

In general, registers in Statistics Denmark are considered to be valid sources of information for epidemiological studies [17,29–32]. Another strength of this study is that a diagnosis of CO-poisoning is simple and therefore limits misclassification. Usually, an arterial blood sample is analyzed for HbCO, and levels above 3% indicate intoxication (7–9% for smokers) [33]. A total of 40.0% of the patients who died from CO-poisoning since 2002 and underwent autopsy, making the cause of death for this group even more accurate.

In the present study, we decided to split patients into four age groups, aware that group four would contain patients of a broad range of ages ranging from 55–85 years of age and that patients of higher age may fare differently from younger patients. However, the number of patients in the highest age range was relatively low.A major limitation of this study is the absence of clinical information, especially regarding the severity of CO-poisoning which may have caused some degree of residual confounding in our model.

Different propensity score techniques may be used to estimate treatment effects in observational studies trying to eliminate the impact of observed baseline covariates such as e.g. treatment-selection bias. Inferences about treatment effect using propensity-score matching are only valid if, in the matched sample, treated and untreated subjects have similar distributions of measured baseline potentially confounding factors i.e. covariates. As we do not believe that we would be able to balance measured covariates adequately, we did not apply any propensity score models in the present study in addition to the statistical methods described. Furthermore, causal claims based on propensity score models rely on an assumption, that there are no unmeasured confounders once we condition on the measured covariates. Hidden treatment-selection bias cannot be excluded in the present study.

Furthermore, a diagnosis of CO-poisoning has not been validated in the DNPR. However, we have no reason to believe that this diagnosis in the registry should be less valid than other diagnoses, which have been proven to be valuable for epidemiological research [17,29–32]. Additionally, residual confounding of unmeasured and unknown data cannot be excluded.

## Conclusions

Poisoning from smoke and/or CO is a frequent occurrence in Denmark, accounting for numerous contacts with hospitals and numerous deaths every year. Suicide and suicide attempts contribute heavily to these numbers. Both intoxication and mortality are highly associated with co-morbidities, especially conditions that might interfere with cognitive and physical function. Treatment with HBO is not associated with an effect on short-term or long-term survival after adjusting for CO-morbidities and age.
